# Regulation of Lymphocyte Function by PPAR*γ*: Relevance to Thyroid Eye Disease-Related Inflammation

**DOI:** 10.1155/2008/895901

**Published:** 2008-03-02

**Authors:** G. M. Lehmann, T. M. Garcia-Bates, T. J. Smith, S. E. Feldon, R. P. Phipps

**Affiliations:** ^1^Department of Environmental Medicine, University of Rochester School of Medicine and Dentistry, University of Rochester Medical Center, Rochester, NY 14642, USA; ^2^Department of Microbiology and Immunology, University of Rochester School of Medicine and Dentistry, University of Rochester Medical Center, Rochester, NY 14642, USA; ^3^Division of Molecular Medicine, Harbor-UCLA Medical Center, California Los Angeles Medical Center, Torrance, CA 90502, USA; ^4^Jules Stein Eye Institute, University of California Los Angeles, Los Angeles, CA 90095, USA; ^5^David Geffen School of Medicine, University of California Los Angeles, Los Angeles, CA 90095, USA; ^6^University of Rochester Eye Institute, University of Rochester Medical Center, Rochester, NY 14642, USA; ^7^Lung Biology and Disease Program, University of Rochester School of Medicine and Dentistry, University of Rochester Medical Center, Rochester, NY 14642, USA

## Abstract

Thyroid eye disease (TED) is an autoimmune condition in which intense inflammation leads to orbital tissue remodeling, including the accumulation of extracellular macromolecules and fat. Disease progression depends upon interactions between lymphocytes and orbital fibroblasts. These cells engage in a cycle of reciprocal activation which produces the tissue characteristics of TED. Peroxisome proliferator-activated receptor-*γ* (PPAR*γ*) may play divergent roles in this process, both attenuating and promoting disease progression. PPAR*γ* has anti-inflammatory activity, suggesting that it could interrupt intercellular communication. However, PPAR*γ* activation is also critical to adipogenesis, making it a potential culprit in the pathological fat accumulation associated with TED. This review explores the role of PPAR*γ* in TED, as it pertains to crosstalk between lymphocytes and fibroblasts and the development of therapeutics targeting cell-cell interactions mediated through this signaling pathway.

## 1. INTRODUCTION

Peroxisome proliferator-activated receptors (PPARs) are members of the nuclear hormone receptor superfamily that bind to DNA as heterodimers formed with retinoid X receptors (RXRs) [1]. These heterodimers control gene expression by binding to a specific *cis* acting DNA element known as the peroxisome proliferator response element (PPRE) found in the promoter or enhancer regions of target genes. PPRE binding can occur in the presence or absence of ligand and can either induce or repress gene transcription in a cell-specific manner. The ability of PPAR-RXR heterodimers to transactivate genes results not only from their binding to DNA, but also from their association with transcriptional coactivators or corepressors. Usually, agonist binding to these receptors inhibits corepressor and promotes coactivator binding, resulting in increased transcription of target genes.

Three PPAR subtypes, PPAR*α* (NR1C1), PPAR*β*/*δ* (NUC1, NR1C2), and PPAR*γ* (NR1C3), are encoded by separate genes [2]. Three isoforms of PPAR*γ*, PPAR*γ*1, PPAR*γ*2, and PPAR*γ*3 are generated by alternative splicing of the same mRNA [3]. PPARs are differentially expressed in a variety of tissues and are important to the regulation of lipid and carbohydrate metabolism, energy homeostasis, cellular differentiation, apoptosis, and immunity and inflammatory responses [2, 4–6]. The physiological functions of PPAR*α* and PPAR*γ* have been well characterized, whereas the physiological function of PPAR*β*/*δ* is poorly understood although the protein is widely distributed [3]. PPAR*α* is expressed in brown adipose tissue, liver, kidney, heart, and skeletal muscle, but is also detected in cells of the vasculature and the immune system [1, 3, 7–10]. Its activation affects transcriptional expression of many genes involved in fatty acid oxidation, lipid metabolism, and inflammation [8, 11]. PPAR*α* agonists (including the fibrates) have been reported to increase levels of high-density lipoproteins (HDL), lower those of triglycerides and decrease weight gain [12, 13]. They also induce adipogenesis in fibroblasts in vitro through the induction of genes such as high-mobility group AT-hook 2 (*HMGA2*) and leptin [8, 14–18].

PPAR*γ* is highly expressed in adipose tissue, colon, retina, and in cells of the immune system, including platelets [1, 3–5, 19–25]. The PPAR*γ*1 isoform is the more widely expressed, while PPAR*γ*2 is mainly found in adipose tissue and liver [3, 26]. PPAR*γ*3 mRNA is detectable in mouse macrophages, but little is known about the protein expression and functional significance of this isoform [3, 27]. Synthetic PPAR*γ* agonists, including drugs of the thiazolidinedione (TZD) family (e.g., ciglitazone, pioglitazone, rosiglitazone and troglitazone), have potent insulin-sensitizing properties [3, 28, 29]. Because of this, rosiglitazone and pioglitazone are often prescribed for the treatment of type 2 diabetes mellitus [3]. These and naturally occurring PPAR*γ* ligands, such as lysophosphatidic acid [30], nitrolinoleic acid [31], prostaglandin D_2_(PGD_2_), and 15-deoxy-Δ^12,14^-prostaglandin J_2_(15d-PGJ_2_) [32, 33], are also potent promoters of adipogenesis [3, 28, 34–37]. PGD_2_ and 15d-PGJ_2_ are derived from arachidonic acid by the catalytic activities of the cyclooxygenase-2 (Cox-2) and prostaglandin D synthase enzymes [28, 32, 33]. PGD_2_ spontaneously undergoes a series of dehydration reactions to form the PGJ family of prostaglandins, including 15d-PGJ_2_, and 15d-PGD_2_, which can also transactivate PPAR*γ* and induce adipogenesis [28, 38–41]. Many of the genes under PPAR*γ* control are important to glucose uptake, lipid metabolism and storage, as well as adipogenesis, explaining the ability of PPAR*γ* ligands to increase insulin sensitivity and to trigger the differentiation of fibroblasts to adipocytes [8, 42–44]. Others act to dampen inflammation by decreasing TNF*α*, IL-6, and IL-8 production, suggesting potential therapeutic applications in chronic inflammatory diseases [45]. It has been suggested that the adipogenic action of PPAR*γ* could serve as another of its anti-inflammatory functions because remodeling of inflamed tissue to fat may render it more quiescent [28]. Others would argue that adipogenesis is a proinflammatory action because an increase in fat mass would result in increased release of proinflammatory adipocytokines [36]. In any case, increased adipogenesis may lead to disease, even if it serves to attenuate active inflammation. Thyroid eye disease (TED) provides a cogent example of such a circumstance. This review will explore the role that PPAR*γ* and lymphocytes play in advancing pathological tissue remodeling in TED and how PPAR*γ* may be exploited as a target for therapeutic strategies.

## 2. THYROID EYE DISEASE

TED is a condition in which intense inflammation leads to remodeling and expansion of the connective and adipose tissues of the orbit, including proliferation and differentiation of fibroblasts to adipocytes, fat deposition, and disordered accumulation of extracellular matrix glycosaminoglycans (GAGs) [8, 46, 47]. Accumulation of GAGs is accompanied by dramatic swelling due to their prodigious water-binding capacity [48, 49]. The increased volume of orbital connective tissue leads to forward protrusion of the eye (exophthalmos), accompanied by nerve and muscle damage [28, 50–56]. In patients with severe TED, the initial inflammation subsides, but infiltration of muscle fibers by fibroblasts leads to fibrosis, potentially limiting their motility [46, 47, 50–52]. In addition to exophthalmos and extraocular muscle dysfunction, clinical features of TED include periorbital edema, eyelid retraction, dry eye, pain, optic neuropathy, double vision, and vision loss [28, 50, 53, 57].

TED is closely associated with Graves' disease (GD), a common autoimmune disorder in which stimulatory autoantibodies against the thyroid-stimulating hormone receptor (TSH-R) cause the thyroid to produce excess thyroid hormone [50, 54, 58, 59]. In addition to the hypermetabolic consequences of hyperthyroidism, clinically apparent TED develops in approximately 50–60% of patients with GD [50, 54–56]. Furthermore, a subset of patients with severe TED develop pretibial dermopathy, a distinctive thickening of the skin, usually occurring on the anterior lower leg [60, 61]. Although the pathogenesis of the hyperthyroid state in GD is relatively well understood, many questions remain regarding the induction and perpetuation of the orbital (and pretibial) disease that develops in some patients. It is likely that the hyperthyroid state does not promote connective tissue accumulation within the orbit. Euthyroid GD patients remain at risk for developing TED [62, 63]. Furthermore, TED does not usually occur in patients with non-Graves’ hyperthyroidism [64]. It has been suggested that the orbit is a secondary target of autoimmune attack, involving the same autoantigen (TSH-R), but resulting in consequences distinct from those in the thyroid [50, 58, 65]. However, TSH-R mRNA and protein are expressed widely in many tissues which are unaffected in GD, so the basis for the anatomical restriction of TED remains unclear [50, 66]. Moreover, no convincing evidence currently exists for TSH-R mediating any important biological events in orbital connective tissues.

To date, there are no effective means of preventing the onset of TED or for predicting which GD patients are likely to exhibit extrathyroidal complications. A study by Khoo et al. [67] suggested that the presence of thyroid-stimulating antibodies combined with the absence of antibodies against thyroid peroxidase is a predictor, but other reports contradict these findings [68, 69]. Current treatment options for TED exist, including corticosteroid treatment, external beam radiation, and surgery, but these interventions are aimed only at the consequences of the disease, and they fail to prevent or reverse pathological alteration of orbital tissues [70]. Histological examination of orbital tissue in TED suggests that its development and progression involve interactions between lymphocytes and fibroblasts [28]. Understanding these complex interactions may both lead to the identification of biomarkers predictive of advanced disease and provide effective early treatments. It is thought that autoreactive B lymphocytes initiate the disease state by producing antibodies against self-antigen, such as the TSH-R [58]. Next, in a poorly understood and likely variable event, autoantibody production results in orbital fibroblast activation [71]. Activated fibroblasts release chemoattractants that recruit T lymphocytes and monocytes to the orbit [28, 37, 50, 72–77]. These bone marrow-derived cells cooperate with the resident fibroblasts and are engaged in a cycle of reciprocal activation which ultimately produces the pathological changes in the orbit characteristic of TED [50].

## 3. INTERACTIONS BETWEEN LYMPHOCYTES AND FIBROBLASTS

Orbital tissue from patients with TED is infiltrated by T helper type I (Th1) and T helper type 2 (Th2) lymphocytes, B lymphocytes, mast cells, and macrophages [47, 50, 59, 78–82]. It is currently thought that these cells, once recruited to the orbit, generate cytokines which participate in driving tissue reactivity and remodeling. Autoimmune responses, like that found with TED, are governed primarily by the actions of B and T lymphocytes. Lymphocytes are migratory cells that proliferate extensively and develop into activated effector cells when they encounter specific antigen in the proper costimulatory context. Normally, the antigens to which lymphocytes respond are foreign and several tolerance mechanisms act to prevent the development of reactivity to self antigens or autoimmunity [83, 84]; but these tolerance mechanisms sometimes fail and autoimmunity develops. B lymphocytes are key to this phenomenon, as activated autoreactive B lymphocytes produce autoantibodies and are a critical source of support for the function of other immune cells, such as T lymphocytes and fibroblasts [85].

Fibroblasts were once viewed as merely structural bystanders in the cellular microenvironment, producing extracellular matrix components, but otherwise uninvolved in the regulation of tissue homeostasis. Now, it is understood that fibroblasts are a highly interactive cell type, described as “sentinel cells,” which are able to detect events that endanger homeostasis, to communicate these dangers to cells of the immune system, and to respond directly to these threats via proliferation and differentiation to effector cells that support tissue integrity [58, 66, 72]. Fibroblasts do not merely respond to immune stimulation, but actively participate in the inflammatory pathway through the synthesis of proinflammatory mediators, including IL-1, IL-6, and IL-8 [28, 73, 74]. They interact with bone marrow-derived cells in the orbit and are key to the pathophysiology of TED [8, 37, 50, 65, 72, 73, 75, 76]. As described earlier, the clinical symptoms of TED result from excess extracellular macromolecular deposition, fibrosis, and fat accumulation in the orbit [48, 57]. Several differences have been identified that distinguish orbital fibroblasts harvested from patients with TED from those derived from normal orbital tissues and nonorbital anatomic sites. Orbital fibroblasts from patients with TED synthesize excess GAGs, including hyaluronan, are unusually proliferative and can differentiate into adipocytes, leading to accumulation of fat [50, 86, 87]. In addition, they do not express IL-1 receptor antagonist at levels found in other fibroblasts. This results in excessively high levels of Cox-2 and PGE_2_ in response to proinflammatory cytokines [47, 50, 59, 77, 86, 88–91]. They also display lymphocyte costimulatory molecules such as CD40 [59, 77, 86, 88]. These characteristics suggest that the fibroblast phenotype underlies the selective anatomic distribution of TED-associated inflammation and tissue remodeling [37, 47, 50, 59, 73, 75, 92, 93].

**Figure 1:**
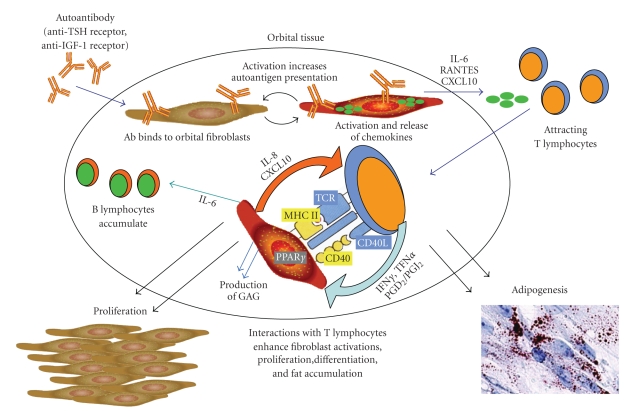
According to one current model, TED is triggered by binding and activation of orbital fibroblasts by autoantibodies. These autoantibodies could be specific for antigens such as TSH-R and/or IGF-1R. Activated orbital fibroblasts release chemokines, including IL-16, RANTES, and CXCL10, which recruit T lymphocytes into the orbit. These lymphocytes then interact with fibroblasts, potentially activating each other, further promoting cytokine production (IFN*γ*, TNF*α*, PGD_2_, and 15d-PGJ_2_) and secretion of T cell-activating factors by the fibroblasts (IL-8 and CXCL10). Fibroblasts are also stimulated to secrete IL-6 (promoting B cell differentiation) and to increase autoantigen presentation, both of which amplify the overall response. The interactions of fibroblasts with T cells result in the deposition of extracellular matrix molecules, fibroblast proliferation, and fat accumulation.

The unique features of orbital fibroblasts provide an environment in which TED might develop, but the disease is characterized also by mononuclear cell infiltration [48, 59, 94]. Substantial data support the concept that infiltrating T lymphocytes interact with fibroblasts, activate them, and result in their proliferation, synthesis of extracellular macromolecules, and differentiation to adipocytes [50, 59]. A summary of this model for the pathogenesis of TED is depicted in Figure 1. It is thought that autoantigen expression by orbital fibroblasts instigates T lymphocyte recruitment to the orbit [48, 95, 96]. The autoantigen may be TSH-R or another protein, such as insulin-like growth factor-1 receptor (IGF-1R) [34, 48, 54, 94–98]. Recruited T lymphocytes stimulate orbital tissue remodeling by initiating fibroblast proliferation and hyaluronan synthesis [50]. They also contribute to the perpetuation of the inflammatory response by (1) stimulating fibroblast production of chemokines, like IL-16 and RANTES, and cytokines, like IL-6, that initiate T and B lymphocyte migration to local environments, and (2) increasing fibroblast presentation of autoantigens [50, 73, 74, 76, 77, 99]. The T lymphocyte-fibroblast interaction occurs via costimulatory molecules, adhesion molecules, and cytokines like IFN*γ*, IL-1*β*, and TNF*α* [50, 99]. One mechanism by which T lymphocytes may communicate with orbital fibroblasts is through the CD40-CD40 ligand pathway [50, 74, 88]. CD40 is a cell surface receptor found on antigen-presenting cells, whereas CD40 ligand (CD40L, CD154) is expressed on T lymphocytes [50]. Ligation of CD40 on B lymphocytes or other antigen-presenting cells is necessary for efficient activation of T-lymphocyte effector functions [100, 101]. Recently, it has been shown that orbital fibroblasts from TED patients express high levels of CD40, which is upregulated in the presence of IFN*γ*, produced by infiltrating T lymphocytes [74, 76, 77, 99]. Activation by CD40L induces hyaluronan synthesis, IL-6 and IL-8, Cox-2 and PGE_2_ [50, 74, 86, 102]. Thus, the CD40-CD40L bridge is one potential pathway through which T lymphocytes could influence fibroblast activation and proliferation in TED [50].

Fibroblasts respond to T lymphocyte-mediated activation by releasing factors that recruit, activate, and promote the proliferation of T lymphocytes, thus participating in the perpetuation of inflammation [35, 50, 103]. In patients with clinically significant TED, even in those whose hyperthyroidism is well controlled, B and T lymphocytes have been shown to display a distinctly activated phenotype different from those derived from control donors [59]. This sustained activation following treatment of hyperthyroidism contributes to orbital inflammation and tissue remodeling observed in late-stage TED. A recent study found that orbital fibroblasts from TED patients may modulate the activity of T lymphocytes through the production of CXCL10 [35]. TED patients with active disease had higher serum CXCL10 levels than patients with inactive disease. CXCL10 release enhances the migration of T lymphocytes into the orbit, where they secrete IFN*γ* and TNF*α*. IFN*γ* levels were higher in TED patients than in patients with GD without orbital involvement. IFN*γ* and TNF*α* synergistically induced CXCL10 release by orbital fibroblasts, thereby perpetuating a positive feedback loop [35, 50, 103]. PPAR*γ* activation was found to play an inhibitory role in this process, both in vivo and in vitro [35].

## 4. PPAR*γ* LIGANDS AND INFLAMMATION

PPAR*γ* ligands attenuate activity of inflammatory bowel disease in animal models [35, 104–106], experimental autoimmune encephalomyelitis [107, 108], arthritis [21], and psoriasis [109]. Clinical trials have shown that they ameliorate inflammation in patients with mild-to-moderate cases of ulcerative colitis [1, 110, 111]. At least some of the anti-inflammatory effects of PPAR*γ* ligands result from direct actions on cells of the innate and adaptive immune system [23, 112–114]. In macrophages, they inhibit activation and production of inflammatory cytokines such as TNF*α*, IL-1*β*, and IL-6 [25, 115, 116]. In addition, PPAR*γ* activation has been shown to skew macrophage differentiation into a more anti-inflammatory phenotype [117]. In dendritic cells, PPAR*γ* agonists downregulate the synthesis of chemokines involved in the recruitment of T lymphocytes [35, 118].

Evidence for a physiological role of PPAR*γ* in regulating B lymphocyte function was generated in studies using PPAR*γ*-haploinsufficient mice [21]. B lymphocytes derived from these mice exhibit increased proliferation and survival, enhanced antigen specific immune response, and spontaneous NF-*κ*B activation [1, 21]. Our laboratory has shown that normal and malignant mouse and human B lymphocytes express PPAR*γ* and that exposure to certain PPAR*γ* ligands inhibits their proliferation and can induce apoptosis [24, 113, 119]. Several anti-inflammatory mechanisms of PPAR*γ* have been suggested, including inhibition of NF-*κ*B, AP1 and STAT transcription factors [120, 121]. A recent study demonstrated that some of these effects are PPAR*γ*-independent [122]. PPAR*γ* also regulates inflammation by blocking gene transcription through “transrepression.” Several models of transrepression by PPAR*γ* have been proposed. In one of them, PPAR*γ*-RXR complexes are thought to sequester coactivators, thereby downregulating other transcription factors. A second model suggests that interactions between transcription factors result in mutual antagonism of gene activation [123]. A recent report by Pascual et al. demonstrated a PPAR*γ* ligand-dependent sumoylation of PPAR*γ* that leads to its recruitment to repressor complexes in the promoter regions of inflammatory genes regulated by NF-*κ*B. This prevents their release and suppresses proinflammatory gene expression [124].

PPAR*γ* also plays a role in T lymphocyte regulation, and its level is upregulated following activation [5, 125]. PPAR*γ* ligands inhibit T lymphocyte proliferation and reduce the production of IFN*γ*, TNF*α*, and IL-2 [23, 126, 127]. These inhibitory effects result from the direct interaction between PPAR*γ* and the transcription factor nuclear factor of activated T cells (NFAT) [128]. Recent observations reported by Wohlfert et al. could illuminate yet another mechanism through which PPAR*γ* controls immune responses [129]. They investigated the connection between PPAR*γ* and CD4^+^ CD25^+^ regulatory T lymphocytes (Tregs). Tregs have been demonstrated to play a key role in regulating autoimmunity and immune responses [130–132]. There are two different subtypes of Tregs: thymus-derived natural Tregs (nTregs) and inducible or adaptive Tregs (iTregs). nTregs are always present in normal individuals as a functionally mature population constitutively expressing CD25, while iTregs are CD4^+^ CD25^+^ T lymphocytes which differentiate from CD4^+^ CD25^−^ effector T lymphocytes in the periphery under a specific cytokine stimulation [133, 134]. Wohlfert et al. showed that ciglitazone enhanced the conversion of effector T lymphocytes into iTregs. Moreover, PPAR*γ* expression in nTregs was required for the in vivo effects of ligand treatment in a murine model of graft-versus-host disease. These findings suggest that PPAR*γ* ligands may enhance the activity of regulatory T lymphocytes while dampening the activation of other T lymphocyte subsets. The anti-inflammatory potential of PPAR*γ* may be relevant to TED because this transcription factor is present in orbital tissues from TED patients, its activity may be involved in the regulation of IFN*γ*-induced chemokine expression, and its activators might attenuate the recruitment of activated T lymphocytes to sites of inflammation [35, 106, 118, 135, 136]. Together, the evidence indicates that PPAR*γ* ligands could interrupt communication between mononuclear cells and fibroblasts [1, 35, 50]. However, PPAR*γ* ligands may also promote T lymphocyte synthesis of IL-8 [137, 138]. Thus, the effects of PPAR*γ* on T lymphocytes are complex and require further study.

**Figure 2:**
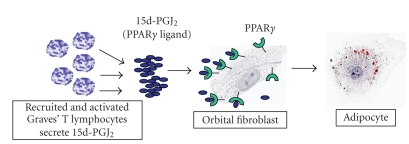
T lymphocytes in TED patients express constitutively elevated levels of Cox-2, one enzyme critical to the production of the naturally-occurring PPAR*γ* ligand 15d-PGJ_2_. When these lymphocytes infiltrate the orbit, 15d-PGJ_2_ is secreted in resident fibroblasts result in their differentiation into adipocytes.

End-stage TED can culminate with permanent pathological changes including the differentiation of fibroblasts to adipocytes that contribute to increased connective tissue volume [28]. Adipogenesis is regulated by the interplay of several factors, including PPAR*α* and *γ* [8, 28, 42, 139]. Natural and synthetic activators of PPAR*γ* are known to stimulate lipid accumulation and the expression and secretion of adiponectin [28, 34, 139, 140]. PPAR*γ* antagonists prevent triglyceride accumulation in orbital fibroblasts exposed to PPAR*γ* agonists. This supports the concept that PPAR*γ* expression and activation are crucial for adipocytic differentiation [28, 35, 36]. PPAR*γ* levels are higher in orbital tissue from patients with active TED than in controls or individuals with inactive TED [35, 135]. Responses of orbital fibroblasts to PPAR*γ* ligands provide an interesting link to T lymphocyte activity. T lymphocytes from patients with GD express constitutively high levels of Cox-2, and produce substantial PGD_2_ and 15d-PGJ_2_ [28, 141]. We have developed the model depicted in Figure 2, in which T lymphocyte infiltration of the orbit results in adipocytic differentiation of fibroblasts [28, 142]. In fact, coculture of orbital fibroblasts from TED patients with activated T lymphocytes results in cytoplasmic accumulation of lipid droplets in fibroblasts [28].

## 5. PPAR*γ* AND TISSUE REMODELING

Adipogenesis has been suggested to be a mechanism for stanching chronic inflammation [28]. Alternatively, this process may promote further inflammation by increasing proinflammatory adipocytokine production [36]. Orbital adipocytes express immunoreactive and functional TSH-R [8, 34, 54, 87, 95, 97, 98]. Positive correlation between TSH-R, PPAR*γ*, and other adipocytic differentiation markers has been observed in tissues from TED patients [34]. Upregulation of an autoantigen on the surface of orbital fibroblasts could enhance the recruitment of autoreactive T lymphocytes to the orbit, fueling inflammation [36, 55]. Whether adipogenesis serves to abate or amplify inflammation, the associated increase in orbital tissue mass is undesirable. Thus, despite anti-inflammatory actions of PPAR*γ*, its proadipogenic functions in the orbit might worsen the disease, contraindicating the use of agents activating this pathway in TED [36]. Several case reports have described development of exophthalmos in patients receiving TZD treatment for type 2 diabetes [28, 36, 143]. In particular, a patient with stable and inactive TED experienced aggravated disease with orbital fat expansion following pioglitazone therapy [28, 35, 36].

## 6. PPAR*γ* AS A THERAPEUTIC TARGET

PPAR*γ* modulators with selective activities would be required if PPAR*γ* function is to be targeted as a TED therapeutic. Identification of selective PPAR*γ* modulators, or SPPAR*γ*Ms, has been sought as a better therapy for type 2 diabetes [3, 144]. In this context, designing partial PPAR*γ* agonists that display insulin-sensitizing activity but lack adipogenic properties might be attractive [3, 144, 145]. The SPPAR*γ*Ms take advantage of both the large ligand-binding domain of PPAR*γ* and the complex interactions between PPAR*γ* and its coactivators and corepressors [1, 3, 144, 146]. The ligand binding domain mediates interactions with transcriptional coactivator or corepressor proteins through ligand-dependent conformational changes in the C-terminal activation function 2 (AF2) *α*-helix [1, 144, 146]. In the absence of ligand, PPAR*γ* functions as an active transcriptional repressor by binding both target genes and transcriptional corepressors [1]. Binding of classical ligands causes the AF2 *α*-helix to move in such a way that a high-affinity binding site for nuclear receptor coactivator proteins is created while corepressor proteins are dislodged from their binding sites [1, 144, 146–149]. Therefore, the structural change in AF2 resulting from agonist binding serves to both inhibit corepressor interaction and promote coactivator recruitment [1]. Because the position of the AF2 domain relative to the ligand binding domain determines whether coactivators or corepressors are recruited, ligands that fit into the binding domain without directly interacting with the AF2 helix, such as SPPAR*γ*Ms, can act as agonists for some receptor functions and as antagonists for others [1, 3, 144, 145, 150–153].

Although not yet clinically available, several SPPAR*γ*Ms have shown promise as potential glucose-lowering agents in type 2 diabetes. For example, metaglidasen has been shown *in vitro* to act as a partial PPAR*γ* agonist/antagonist, with only a weak ability to recruit coactivators, such as CBP, DRIP205/TRAP220, and p300 [144]. Compared to rosiglitazone, metaglidasen is less adipogenic in primary human adipocytes and mouse 3T3-L1 adipocytes. In rodent models of insulin resistance, both metaglidasen and another SPPAR*γ*M, PAT5A, increased insulin sensitivity to levels comparable to those seen with rosiglitazone, with only weak adipogenic potential [3, 144, 154]. Consistent with the preclinical findings, metaglidasen appears to have comparable efficacy to pioglitazone and rosiglitazone in type 2 diabetics, without the undesirable side effect of weight gain [144]. Since developing SPPAR*γ*Ms to target insulin resistance seems achievable, it is anticipated that the anti-inflammatory properties of PPAR*γ* will be targeted in the future [3].

## 7. FUTURE PROSPECTS

PPAR*γ* may play an important role in the development of TED. Studies have taken advantage of the availability of orbital tissue from TED patients. Orbital tissues from patients with GD but without TED are far less available. Potential differences between orbital tissues from “normal” and TED patients have not been fully explored. Similarly, few comparisons between tissues from early and late stage TED patients have been possible. Thus, an animal model of TED with fidelity to human disease is critical.

T lymphocytes and fibroblasts exist as multiple phenotypic subsets in the orbit. Aniszewski et al. [82] found that the phenotypes of orbital T lymphocytes in TED patients changed with disease duration. From that report, the T helper lymphocyte Th1 subset may predominate early, while Th2 lymphocytes may become more abundant later. Furthermore, as discussed previously, the role of Tregs in TED may differ from that of Th1 and Th2 lymphocytes. Studies comparing PPAR*γ* expression and function in each of these subpopulations may lead to better understanding of the role that this transcription factor plays in TED.

Like T lymphocytes, orbital fibroblasts exist in multiple subpopulations. Two major subsets of orbital fibroblast are defined based on their expression of a surface protein known as Thy-1 (CD90) whose function is unknown [37, 73, 155, 156]. The balance between Thy-1 negative and Thy-1 positive populations in the orbit may prove important to normal regulation of inflammation because these subsets exhibit distinct biosynthetic capabilities [73]. However, this balance may also be critical to the development and progression of TED. Depending on the signaling environment and their phenotype, fibroblasts can be stimulated to differentiate into myofibroblasts or lipofibroblasts [37, 157]. Myofibroblasts are important in wound healing, but they may also contribute to fibrosis in late-stage TED patients [158]. The presence of lipofibroblasts is an indication of pathology; in TED, their presence may result in excess orbital fat deposition [28]. Data suggest that the potential for terminal differentiation depends on Thy-1 display. TGF-*β* triggers differentiation of Thy-1^+^ fibroblasts into myofibroblasts, identified by their expression of *α*-SMA [157]. Adipocytic differentiation occurs in the Thy-1^−^ subset [37, 157]. PPAR*γ* expression or function may differ between Thy-1^+^ and Thy-1^−^ subsets, explaining their divergent potential for differentiation.

Finally, TED is one of several pathological conditions in which chronic inflammation leads to tissue remodeling and inappropriate fat deposition. Sjögren syndrome, inflammatory bowel disease, nonalcoholic fatty liver disease, and atherosclerosis are examples [159–162]. PPAR*γ* has been shown to play a major role in the regulation of atherogenesis by countering the inflammation-provoking action of platelet adhesion and activation [3]. Because PPAR*γ* has been implicated in these diseases, it may prove an important determinant in diseases such as TED.
